# Idiopathic scrotal hematoma in a neonate

**DOI:** 10.3205/000288

**Published:** 2021-02-19

**Authors:** Sylvia Gkantseva-Patsoura, George Katsaras, Petroula Georgiadou, Nektarios Lainakis, Eirini Liovarou, Rita Theofanopoulos, Martha Theodoraki

**Affiliations:** 1 Neonatal Intensive Care Unit, General State Hospital of Nikaia “Agios Panteleimon”, Piraeus, Greece; 2 Paediatric Department, General Hospital of Pella – Hospital Unit of Edessa, Greece; 3 Paediatric Surgery Department, General State Hospital of Nikaia “Agios Panteleimon”, Piraeus, Greece; 4 Radiology Department, General State Hospital of Nikaia “Agios Panteleimon”, Piraeus, Greece

**Keywords:** neonate, scrotal hematoma, idiopathic

## Abstract

**Background:** Neonatal scrotal hematoma is considered a surgical emergency in the neonatal period. Up to recently, immediate surgical exploration was considered the gold standard for the diagnosis and treatment in the underlying causes.

**Objective:** In this article, we present a case of idiopathic scrotal hematoma in a neonate.

**Method:** It was managed conservatively with clinical and ultrasonographic follow-up.

**Result:** The hematoma had gradually subsided, and any surgical intervention was avoided to the neonate.

**Conclusion:** With good clinical and imaging follow-up, some cases could be managed nonoperatively.

## Zusammenfassung

**Hintergrund:** Das neonatale Skrotalhämatom wird als chirurgischer Notfall in der Neugeborenenperiode angesehen. Bis vor Kurzem wurde die sofortige chirurgische Untersuchung als Goldstandard für die Diagnose und Behandlung der zugrunde liegenden Ursachen angesehen.

**Ziel:** In diesem Artikel stellen wir einen Fall eines idiopathischen Skrotalhämatoms bei einem Neugeborenen vor.

**Methode:** Es wurde konservativ mit Beurteilung der Klinik und mittels Ultraschallverlaufskontrollen beobachtet.

**Ergebnis:** Das Hämatom war allmählich rückläufig und ein chirurgischer Eingriff bei unserem Neugeborenen wurde vermieden.

**Schlussfolgerung:** Mit einer guten klinischen und bildgebenden Nachsorge könnten einige Fälle nicht operativ behandelt werden.

## Introduction

Scrotal hematoma is the collection of blood inside the scrotum, which contains the testicle, the epididymis and the spermatic cord. Testicular hematomas can form within the testicular parenchyma or on the surface of the testicle. Scrotal hematoma can occur secondary to some intra-abdominal diseases, including intraperitoneal or retroperitoneal bleeding [1]. It is a rare genitourinary emergency in a neonate [2]. Some cases have clear etiology such as testicular torsion, inguinal hernia, adrenal hemorrhage, meconium peritonitis, hematocele, testicular tumor or birth trauma, whereas other cases have no definite cause. Because Doppler ultrasonography (DUS) in the small neonatal testis has previously been regarded as an inconsistently diagnostic method, scrotal exploration was considered necessary for the diagnosis and treatment of these neonates [3], [4]. In this article, we present a case of idiopathic scrotal hematoma in a neonate which was managed conservatively with clinical and radiological follow-up, emphasizing the role of ultrasonography in the evaluation of the several potential causes leading to an acute scrotum, and consequently sparing some neonates surgical exploration.

## Case description

A late preterm male neonate (Ballard score: 36 weeks gestational age) was delivered by a 21-year-old woman at home with no professional care, no labors in pregnancy. The neonate was transported by ambulance to the nearest hospital, where he was resuscitated and intubated. Afterwards, he was again transported by ambulance to our tertiary NICU at approximately the 6^th^ hour of life. Since the neonate was hemodynamically stable at the time of admission, he was extubated. He was a small for gestational age neonate with 2000 gr body weight. The examination of the abdomen was normal. Painless swelling of the scrotum and hydrocele were clinically observed. The neonate had a hematocrit of 48.6%, negative Coombs test and normal values of the coagulation parameters (activated partial thromboplastin time (aPTT), prothrombin time (PT), international normalized ratio (INR), platelet count and fibrinogen). He received vitamin K.

**Figure 1:**
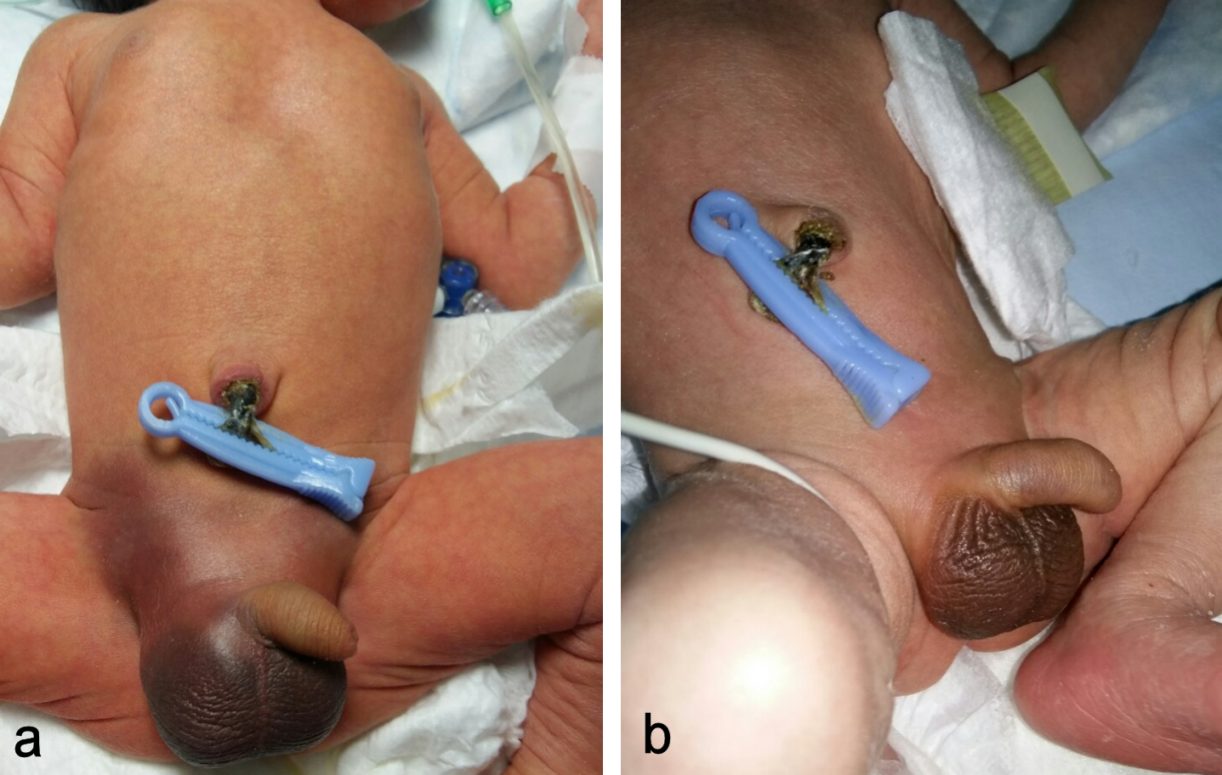
a) At the 3^rd^ day of life, bruises are observed at both groins and the scrotum. b) The same with complete resolution after two weeks.

At the 3^rd^ day of life, jaundice appeared, and phototherapy started. Furthermore, bruises were observed at both groins as well as at the whole scrotum (Figure 1a (Fig. 1)). The swelling of the scrotum was thickened. There was no clinical feature suggestive of trauma or bleeding diathesis. The ultrasonography of the abdomen, the adrenals and the testicles were all normal. Clinical examination by a pediatric surgeon took place that excluded the acute scrotum.

**Figure 2:**
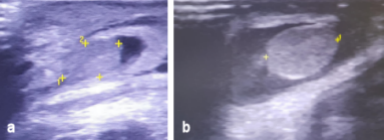
a) Ultrasonography of the right testicle. b) Ultrasonography of the left testicle. Both testicles are depicted inside the scrotum. The scrotal wall as well as the testicles are grossly thickened and edematous, while the echogenic structure and vascularity of the testicles are normal.

At the 4^th^ day of life, the phototherapy was stopped. Due to the progressing clinical condition at the groins and scrotum, we conducted a new ultrasonography at these areas. The scrotal wall was grossly thickened and edematous, while the echogenic structure and vascularity of both testicles were normal (Figure 2a, b (Fig. 2)). Based on the clinical image, the finding concerned a hematoma of the scrotum. In this phase, no hydrocele was observed.

**Figure 3:**
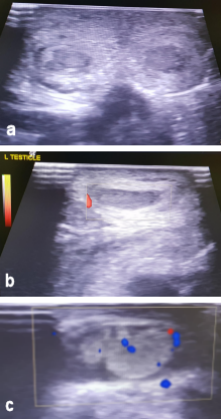
a) Ultrasonography of both testicles. b) Doppler ultrasonography of the right testicle. c) Doppler ultrasonography of the left testicle. Both testicles maintain their normal echogenic structure and vascularity. Thickening of the scrotum wall is still observed in the context of hematoma. A hydrocele with echogenic elements on the left scrotum (hematoma) is observed.

The child was managed non-operatively. He was monitored clinically and radiologically. In the following days, the testicles maintained their normal echogenic structure and vascularity, however, thickening of the scrotum wall was still observed in the context of hematoma, while at the same time a hydrocele with echogenic elements on the left scrotum (hematoma) was observed to be more intensive (Figure 3a, b, c (Fig. 3)). Follow-up ultrasound scans revealed complete resolution of the earlier noted hematoma at the scrotal wall.

## Discussion

Acute scrotal hematoma in the neonate might be a major surgical emergency [5]. Although the surgical exploration was considered the gold standard, clinical evaluation and ultrasonography have been shown to be of greatest value in the differential diagnosis of testicular diseases, as discussed in AWMF guideline 006/23 from 2015 [6], [7].

The most common causes for acute scrotal hematoma include testicular torsion, inguinal hernia, scrotal or testicular edema and scrotal trauma, whereas adrenal hemorrhage and adrenal neuroblastoma are rare but must also be considered. Idiopathic scrotal edema and hydrocele are among the minor causes. Spontaneous idiopathic hemorrhage of the scrotum, as found in our patient, has also been described and is usually a diagnosis of exclusion [1], [8], [9].

Our patient, at the 3^rd^ day of life, presented with bruises at both groins as well as at the whole scrotum. There was no clinical feature suggestive of trauma or bleeding diathesis. The ultrasonography of the abdomen, the adrenals and the testicles were normal.

Ultrasonography, including power Doppler, is usually performed to differentiate conditions such us testicular torsion, scrotal or testicular edema, inguinal hernia, testicular trauma, or adrenal hemorrhage in an acute scrotum. In testicular torsion, there is hypovascularity of the affected testis, while testicular trauma may present with intratesticular hematoma or laceration of the tunica albuginea. Acute scrotal discoloration in neonates is usually caused by testicular torsion [10]. Also few cases of scrotal hematoma in neonates due to adrenal hemorrhage have been reported in the literature [11]. Scrotal and abdominal ultrasonography can provide important information about the patient, and this would seem to be essential in order to avoid unnecessary invasive procedures. Spontaneous hemorrhage of the scrotum, as found in our patient, has also been described in the literature [12].

Except for spermatic cord torsion and inguinal hernia, hemoscrotum due to other causes may be managed with conservative treatment. We treated our patient nonoperatively as well as with systematic clinical and sonographic follow-up examinations, and there was a gradual improvement of the symptoms (Figure 1b (Fig. 1)).

## Conclusion

The diagnosis of neonatal scrotal hematoma should be considered in a newborn with scrotal swelling and bluish discoloration. Our case presentation constitutes further evidence and enhances the importance of Doppler in the indication for surgical exploration. Some risk factors can be identified, e.g. evidence of bleeding diathesis, maternal gestational diabetes and high birth weight, with evidence of delivery-related trauma or predisposing intra-abdominal lesions (adrenal hemorrhage, subcapsular liver hematoma) on ultrasonography. A thorough clinical examination and ultrasonography are the cornerstones in establishing an early diagnosis.

## Notes

### Informed consent

Informed consent has been obtained from the patient’s mother for the publication of this case report.

### Competing interests

The authors declare that they have no competing interests.

### Authors’ contributions

Sylvia Gkantseva-Patsoura and George Katsaras participated in the conceptualization, investigation, methodology, writing and original drafting of the manuscript. Petroula Georgiadou, Nikolaos Lainakis, Eirini Liovarou and Rita Theofanopoulos were responsible for investigation, methodology, validation, editing and reviewing the paper. Martha Theodoraki participated in supervision, editing, reviewing and validating the final version.
